# Changing the focus: Facilitating engagement in physical activity for people living with mild dementia in a local community—Protocol for a pre-post mixed methods feasibility study

**DOI:** 10.1371/journal.pone.0307018

**Published:** 2024-09-13

**Authors:** Den-Ching A. Lee, Michele Callisaya, Claudia Meyer, Morag E. Taylor, Katherine Lawler, Pazit Levinger, Susan Hunter, Dawn Mackey, Elissa Burton, Natasha Brusco, Terry P. Haines, Christina Ekegren, Amelia Crabtree, Lisa Licciardi, Keith D. Hill

**Affiliations:** 1 Rehabilitation Ageing and Independent Living (RAIL) Research Centre, Monash University (Peninsula Campus), Melbourne, Victoria, Australia; 2 National Centre for Healthy Ageing (NCHA), Monash University (Peninsula Campus) and Peninsula Health, Melbourne, Victoria, Australia; 3 Peninsula Clinical School, Central Clinical School, Monash University, Melbourne, Victoria, Australia; 4 Peninsula Health, Melbourne, Victoria, Australia; 5 Bolton Clarke Research Institute, Melbourne, Victoria, Australia; 6 College of Nursing and Health Sciences, Flinders University, Australia; 7 Centre for Health Communication and Participation, La Trobe University, Australia; 8 Falls, Balance and Injury Research Centre, Neuroscience Research Australia, Randwick, New South Wales, Australia; 9 Population Health, Faculty of Medicine and Health, UNSW Sydney, Randwick, New South Wales, Australia; 10 School of Allied Health, Human Services and Sport, LaTrobe University, Melbourne, Victoria, Australia; 11 Wicking Dementia Research and Education Centre, College of Health and Medicine, University of Tasmania, Hobart, Tasmania, Australia; 12 National Ageing Research Institute, Parkville, Victoria, Australia; 13 School of Physical Therapy, University of Western Ontario, London, Canada; 14 Department of Biomedical Physiology and Kinesiology, Simon Fraser University, Burnaby, British Columbia, Canada; 15 Curtin School of Allied Health and enAble Institute, Faculty of Health Sciences, Curtin University, Perth, Western Australia, Australia; 16 Aged & Rehabilitation Division, Monash Health, Melbourne, Victoria, Australia; 17 School of Clinical Sciences at Monash Health, Faculty of Medicine, Nursing and Health Sciences, Monash University, Melbourne, Victoria, Australia; 18 Department of Occupational Therapy, Monash University (Peninsula Campus), Melbourne, Victoria, Australia; Public Library of Science, UNITED STATES OF AMERICA

## Abstract

This study aims to address and improve the low physical activity levels among people with mild dementia by implementing a novel shared decision-making and motivational support program, named "Changing the Focus". It will utilise a pre-post mixed methods approach, aiming to recruit 60 community living older people with mild dementia and their care-partners. The shared decision-making process will involve the person living with dementia, their care-partner, and a research therapist, using a purpose-designed discussion tool including factors such as preferred physical activities, health status, local opportunities and program accessibility. This process aims to identify personalised local physical activity opportunities. Participants will be supported with the help of a research therapist to engage in targeted community-based physical activities for 12-months, to progress towards the recommended physical activity guidelines of 150 minutes per week. The intervention provided by the research therapist will include three home visits (baseline, 6- and 12-months) and seven motivational support phone calls (within the first six months). Research therapists may provide additional home visits and support calls as needed. Primary outcomes include program participation (participants living with dementia continuing with the program after 12-months), total physical activity time per week (measured using the Active Australia Survey at baseline, 6- and 12- months) and program acceptability (assessed through semi-structured interviews with participants, care-partners, referrers, and physical activity providers). Secondary outcomes include physical performance, mental health, wellbeing measures, and impact on care-partners (evaluated through physical tests or validated scales at baseline, 6- and 12-months). Other implementation aspects include reach, maintenance, safety (falls, other adverse events) and an economic evaluation. Results will inform feasibility, potential benefits, and challenges associated with this innovative shared decision-making and supported physical activity program for people living with mild dementia. Findings will guide future large-scale studies and contribute to enhancing physical activity opportunities for this population.

## Introduction

Dementia is a priority health issue of older people globally. The prevalence of dementia in Australia is projected to grow from 459,000 in 2020, to 1,076,000 by 2058 [1]. While the major direct impacts of dementia are cognitive impairment and progressive cognitive decline, onset and progression of dementia are also associated with other health problems that have substantial negative personal and care-partner impacts. Impacts to the person living with dementia include reduced independence, physical function, balance, mobility, reduced community and social participation, poor mental health, and increased falls [2–5]. These impacts result in additional resource needs and economic costs for the individual and their family, as well as to health and care systems [6].

Older people typically have low levels of physical activity participation, with only half of older Australians being sufficiently active according to Australian physical activity guidelines [7]. People living with dementia have even lower physical activity levels, [8–10] and rate of physical activity decline over 12-months is approximately 10% higher for people living with mild dementia than people without cognitive impairment [11]. Further, physical activity reduction post-dementia diagnosis is associated with accelerated cognitive decline [12]. For older people, higher physical activity levels reduce the risk of chronic disease and falls, and improve balance, mobility, function, psychological health, wellbeing and quality of life, and reduce social isolation [13–18]. Systematic review evidence supports that various forms of physical activity can be implemented safely and achieve similar outcomes for people living with dementia, [19, 20] including improved activities of daily living, [21] slowed or delayed decline in cognition, improved structural brain changes (e.g. increased hippocampal volume), and reduced neuro-psychiatric symptoms associated with dementia [19, 22] Physical activity programs that include a balance training component have also been safely implemented in people living with dementia, often with care-partner support or in a supervised setting [23, 24] Despite growing evidence of benefits and safety, there remains no systematic approach for identifying physical activity needs, or promoting physical activity for people living with dementia. A preventive approach is needed to curtail the high and growing impacts associated with dementia, and to improve outcomes for people living with dementia and their care-partners before later stages of dementia, when problems become greater and less amenable to intervention [25].

A number of important factors at an individual, organisational and implementation process level have been reported to contribute to the low uptake and sustained participation in physical activity by people living with dementia [10]. Some of these factors include negative perceptions of physical activity, difficulty finding appropriate activities, lack of referral systems, lack of guidance, lack of personalised physical activity options, limited capacity of the health service system, and poor communication mechanisms [10, 26, 27]. Through a co-design process with stakeholders and consumers that included consideration of barriers and facilitators of physical activity participation for people living with dementia in previous research by our team, a new program, *“Changing the Focus”*, has been co-developed. The “*Changing the Focus”* program is based on a systematic approach to identifying physical activity needs and preferences, while supporting engagement and sustained physical activity participation by people living with mild dementia. It involves a community partnership between local referrers and physical activity providers with people living with mild dementia and their informal care-partners. The program includes periodic input and support by research therapists to ensure progression towards the recommended physical activity guidelines for older adults (150 minutes/week of physical activity), [28] in a sustained manner over a 12-month period.

The implementation and evaluation of the “*Changing the Focus*” program is described in this study protocol. The aims of this study are to implement and evaluate “*Changing the Focus”* to determine: i) program participation at 12-months, ii) total physical activity time per week in the preceding week at baseline, 6- and 12-months, iii) program acceptability by people living with dementia, their care-partners, referrers and physical activity providers, iv) potential benefits in physical performance, mental health, wellbeing, and the impact on care-partners at baseline, 6- and 12-months, and v) factors influencing implementation using the RE-AIM (Reach, Effectiveness, Adoption, Implementation, Maintenance) framework, [29] including safety aspects and an economic evaluation of the cost-effectiveness and cost-utility of the program, from a societal perspective.

## Materials and methods

**Fig 1:**
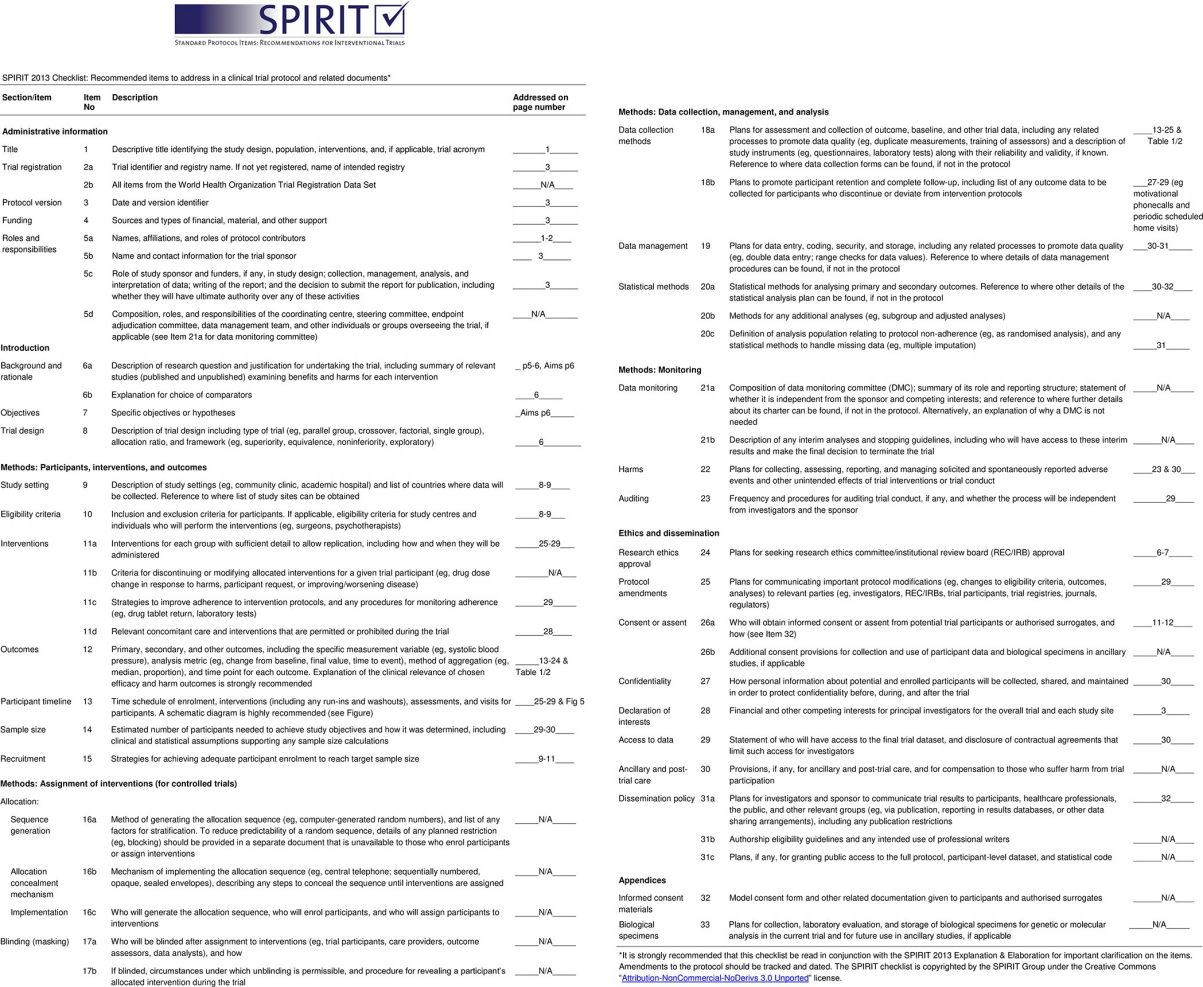
SPIRIT checklist.

The “*Changing the Focus*” study is a single group pre-post mixed methods feasibility study for people living with mild dementia. The study will be conducted in Melbourne, Victoria, Australia. Monash University Human Research Ethics Committee (ID 39672, date 6/11/2023) and Peninsula Health Human Research Ethics Committee (ID 102657, date 4/12/2023) approved the study. The protocol follows the checklist and guidelines described in the SPIRIT guidelines for content of clinical trial protocols (Fig 1) [30] and the ‘Template for Intervention Description and Replication (TIDieR) (S1 Checklist) [31]. Recruitment commenced 4 March 2024.

### Stakeholder and consumer involvement in program development and implementation

#### Preparation work for “*Changing the Focus*”

Stakeholders (a panel of 12 people comprising five physiotherapists and physiotherapy researchers, an exercise physiologist, a geriatrician, two local government representatives, two local physical activity program providers and a Dementia Australia representative) and consumers (four older people and care-partners of people living with dementia) were involved in three parallel meetings to co-design the “*Changing the Focus*” program, (conducted between May and July 2023). Key messages from this co-design phase included: understanding factors influencing past, current and possible future physical activity participation, specific considerations for physical activity providers working with people living with dementia, need for referral pathways, including addressing current barriers to referral, and access and funding constraints.

#### Advisory committee

An Advisory Committee will be established for the project implementation, with six to eight stakeholders, including representative/s from local government, physiotherapist/physical activity program provider/s, two consumers (people living with mild dementia and / or their care-partners), and representative/s from relevant stakeholder organisations. The Advisory Committee will monitor and advise on the conduct of the study, make recommendations for project implementation issues, assist with interpreting study outcomes, and co-develop the dissemination plan for participants and relevant community groups.

### Participants and setting

There are four groups of participants in this study (i) people living with mild dementia, (ii) their care-partners, (iii) referrers of people living with dementia to *“Changing the Focus”*, and (iv) physical activity providers delivering one or more physical activity options to the participants living with dementia.

#### People living with mild dementia

Sixty people living with mild dementia who meet these inclusion criteria will be recruited to the program: adults (age ≥60 years); living in the community or vicinity of Frankston or Mornington Peninsula of Victoria, Australia; have a medical diagnosis of dementia of mild severity [Mini Mental State Examination (MMSE) [32] with education adjustments ≥18], or people with a MMSE score of 18–23 inclusive, at recruitment but who do not have a medical diagnosis of dementia; not housebound due to physical impairments (e.g. severe stroke); not meeting the World Health Organisation physical activity guidelines for older people (<150 minutes moderate/vigorous physical activity/week) [28]; and have capacity to consent, or have a responsible person willing and authorised to provide consent for their participation (see consent details below). People from culturally and linguistically diverse backgrounds who have sufficient English proficiency to understand the study, assessment and intervention instructions, or have a readily available interpreter (family member/friend who will be able to assist) will be included.

#### Informal care-partners of people living with mild dementia

Presence of an informal care-partner (defined as “family member/s and/or friend/s who routinely support the older person through assistance with household tasks, self‐care and mobility, emotional and social support, treatments, medication, responding to acute health needs, advocacy and care coordination, or surrogate decision‐making”) [33] is preferred but not essential. Where available, they will be an important support for the person with dementia during the intervention. Up to 60 informal care-partners who meet these inclusion criteria will be recruited: Adults (age ≥18 years); willing to assist in data collection; and capable to assist the person with dementia to participate in the chosen physical activity program(s) and/or home exercises if required.

#### Referrers of people living with mild dementia

Fifteen to twenty referrers will be recruited (with purposive sampling of health professionals from different practice settings) who meet these inclusion criteria: practice in the community or vicinity of Frankston / Mornington Peninsula regions of Victoria, Australia; have referred one or more clients to the “*Changing the Focus*” study; and willingness to participate in an interview.

#### Physical activity providers of physical activity options for people living with mild dementia

Fifteen to twenty physical activity providers will be recruited (with purposive sampling of physical activity providers from different physical activity options) who meet these inclusion criteria: practice in the community or vicinity of Frankston / Mornington Peninsula regions of Victoria, Australia; have provided a physical activity program attended by one or more of the people living with mild dementia participating in the “*Changing the Focus*” program; and willingness to participate in an interview.

### Recruitment

#### People living with mild dementia

People living with dementia will be recruited from the Frankston and Mornington Peninsula regions of Victoria, using multiple promotional avenues including primary care clinics, allied health private practices, Memory Cafes, local government, promotion through Carers Victoria and Dementia Australia, and the Frankston and the Mornington Peninsula Shire local council newsletters. In addition, recruitment will occur through Peninsula Health’s Cognitive, Dementia and Memory Service (CDAMS), their new Carers Health and Wellbeing Service (commencing in February 2024) and outpatient/community rehabilitation programs. Email and/or telephone contact will be made through the above avenues to introduce the study, and a study flyer circulated to potential participants. Direct recruitment for people living with dementia and their care-partners will also be used (e.g. through advertising on the website of organisations, newsletters, and social media). Referrers (e.g. general practitioners or allied health professionals) from CDAMS/outpatient/community rehabilitation programs/private practice clinics will also be provided with the study flyer, providing details about referral to the study and contact details for the research team.

#### Informal care-partners of people living with mild dementia

Informal care-partners of people living with mild dementia will be recruited through two avenues: (i) where the care-partner may have seen the study promotional materials and initiated contact with the research team for the person living with dementia who they provide care for. At this contact, the care-partner will be asked if they are interested and able to participate in the care-partner component of the study as well; or (ii) where the person with dementia has been referred to the “*Changing the Focus*” study. At the first home visit (to obtain participation consent for the person living with dementia), the research therapist will ascertain if there is an informal care-partner, and if so, will ask if they would be interested in participating in the care-partner component of the project (or organise contact if they are not present). Care-partners’ participation in the study is preferred where available, however if not available, the person living with dementia is still eligible to participate in the study.

#### Referrers

Referrers will be recruited from primary care clinics, allied health private practices, CDAMS, outpatient/community rehabilitation or community health programs, who have referred one or more clients to the “*Changing the Focus*” program. Referrers to the physical activity program may be approached one to two months after making referrals for one or more people living with dementia to the program to undertake an interview with one of the project team with qualitative research experience. Purposive sampling will aim for a diverse mix of referrers (e.g. general practitioners, physiotherapists, exercise physiologists). Selected referrers will be contacted by a research team member by email and/or telephone contact inviting them to participate in the interview.

#### Physical activity providers

Physical activity providers who conduct a physical activity program attended by one or more of the people living with mild dementia participating in the “*Changing the Focus*” program will be recruited through purposive sampling to participate in an interview. Purposive sampling will aim for a diverse mix of providers across different types of physical activity options (e.g. group exercise programs, gymnasium programs, walking programs), as well as those who did and did not participate in an optional training program (providing information about strategies to ensure physical activity programs are provided in a manner to be supportive for people living with dementia to participate, see Intervention section). Selected physical activity providers will be approached by a research team member via phone or email approximately 6-months after one or more people from the *“Changing the Focus”* program commenced their physical activity option/s, inviting them to participate in an interview.

#### Consent and withdrawal process

Participation in this study is voluntary. Following recruitment and screening for eligibility criteria, people living with dementia and their informal care-partners (if applicable) will be provided with an explanatory statement and written consent form for participating in the *“Changing the Focus”* program before an initial home-visit by the research therapist (a physiotherapist or exercise physiologist). At this home visit, the research therapist will determine if the person living with dementia is able to provide consent using a cognitive capacity checklist (see S1 Appendix) [34]. If the person living with dementia is able to provide consent, informed written consent will be obtained. If the person is not capable to give consent, the informal care-partner (or their medical treatment decision maker if they have one) will be asked to sign a separate informed written consent form (providing consent for the person they provide care for). Informal care-partners will provide informed written consent and sign a separate consent form for their involvement in the study. All consent forms will be signed in two copies (one copy for the participant/s to keep and one copy for the research team). For the interviews of people living with dementia and their care-partners, a separate informed written consent form will be used specifically for this component. For both the implementation study and the interview component, people living with mild dementia and informal care-partners can withdraw at any time. Withdrawal from the project will not affect the relationship between the participants and their referrers/physical activity providers, or the project staff.

At each home visit, the research therapist will seek verbal assent from the person living with dementia for their ongoing participation. If there are concerns by the research therapist or the care-partner regarding declined capacity to consent of the person living with dementia subsequent to the initial consent process, the cognitive capacity checklist will be repeated as needed. If the person living with dementia no longer has capacity to consent, alternative consent arrangements (e.g. from the care-partner) will be sought before continuing the program.

Purposefully selected referrers and physical activity providers (see Recruitment section) will be asked to consent to participate in a semi-structured interview. If the referrer or physical activity provider is interested in participating in the interview or finding out more about this component of the study, they will be sent an explanatory statement and written consent form to sign if willing to participate. The signed informed written consent form will be returned to research staff before the interview begins.

### Research therapist recruitment and training

Two to three research therapists (physiotherapists or exercise physiologists) with clinical gerontology experience will be employed for assessments and implementation of *“Changing the Focus”*. They will carry out the home visits, conduct all assessments, discuss with participants regarding suitable physical activity program(s) in the community, refer to or sign participants up for these programs, or provide home-based physical activity for a small number of participants who may prefer this option, and provide motivational supports for participants throughout the program. They will attend an in-person training program detailing the assessment procedures and the intervention, delivered by the chief investigator and/or project manager (who are experienced aged care physiotherapists) and will be provided with written resources (including manual and assessment protocol). They will also participate in the training program for physical activity providers (see Intervention section) that includes details about dementia and its effects, and of considerations in tailoring assessments, interventions, and communication approaches to be suitable for working with people living with dementia. Continual support for the research therapists will be provided by the project manager to ensure consistency of the data collection process and intervention.

### Assessments and outcome measures

**Fig 2:**
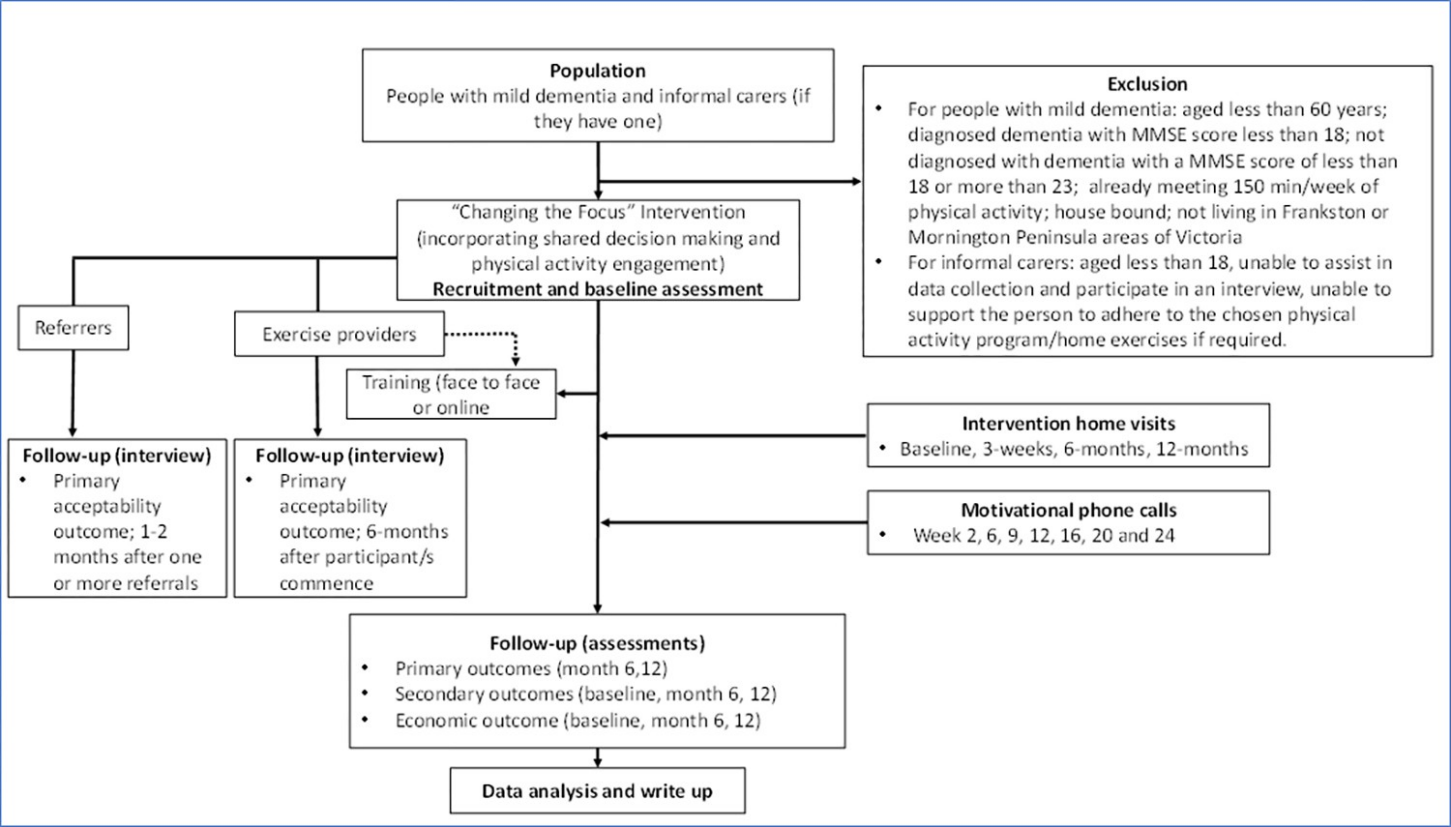
Study flow chart.

Assessments for the person with dementia and their care-partner (if involved) will occur as home visits at baseline, 6-months and 12-months following commencement of the “*Changing the Focus*” program (see Fig 2). Months 6–12 of the program will be predominantly a maintenance phase, without the planned research therapist support. Table 1 provides a summary of study data collected, timing of data collection points, and purpose of data item, and Table 2 provides greater details of the assessment tools and methods for assessment for these measures for people living with dementia.

**Table 1:** Study data collected, timing of data collection points, and purpose of data item.

***For the person with dementia***			
Construct	Measure	Time points (months)	Purpose
Age	Date of birth	0	Describe population
Gender	Categorical	0	Describe population
Dementia diagnosis	Yes/No	0	Describe population
Dementia type	Categorical	0	Describe population
Years living with dementia diagnosis	Years	0	Describe population
Living arrangement	Categorical	0	Describe population
Education level	Categorical	0	Describe population
Medical history	Categorical	0	Describe population
Physical capacity (self-report/observed)	Categorical	0,6,12	Describe population
Use of a walking aid indoors/outdoors	Yes/No	0,6,12	Describe population
Falls	Number • At baseline, the person living with dementia will be asked to recall falls over the last 6-months. • For the following 12-months, the person will complete a falls diary that will prospectively capture the number of falls.	0,6,12	Describe population and analysis of economic efficiency
Falls with injury	Number and open text • At baseline, the person living with dementia will be asked to recall falls and falls injuries, over the last 6-months. • For the following 12-months, the person will complete a falls diary that will prospectively capture the number and injuries from falls.	0,6,12	Describe population and analysis of economic efficiency
***Outcome measures for the person with dementia***			
Program acceptability	Is the “*Changing the Focus*” program suitable for you? • Yes/no/unsure Is the “Changing the Focus” program adequate to meet your needs? • Yes/no/unsure	6,12	Primary outcome
Program acceptability	Semi-structured interview	6 (or at the time of withdrawal)	Primary outcome for implementation evaluation
Total physical activity time / week	Active Australia Survey, time and frequency of physical activity in the preceding week (or most recent “typical” week in the preceding month if the preceding week was not typical for amount of physical activity (e.g. acute health condition)	0, 6,12	Primary outcome
Program participation: Sustained participation in *“Changing the Focus”*	Proportion • The proportion of people living with dementia continuing with their selected program at 12-months	12	Primary outcome
Physical activity: New or modified (e.g increased dosage) of physical activity options undertaken as part of the “*Changing the Focus”* program	Physical activity program and time spent Recorded in exercise diary prospectively	6,12	Describe population, secondary outcome
Life space	Life Space Assessment-Cognitive Impairment (LSA-CI) questionnaire	0,6,12	Describe population, secondary outcome
Dynamic balance	Step test for L leg stepping and R leg stepping	0,6,12	Describe population, secondary outcome
Endurance	2-min walk test	0,6,12	Describe population, secondary outcome
Leg strength	30-second sit-to-stand	0,6,12	Describe population, secondary outcome
Functional mobility	Time-Up-and-Go-test	0,6,12	Describe population, secondary outcome
Cognition	Mini Mental State Examination	0,6,12	Describe population, secondary outcome
Social connectedness	Lubben Social Network Scale-6 (LSNS-6)	0,6,12	Describe population, secondary outcome
Physical activity enjoyment	Physical Enjoyment Scale PACES-8	0,6,12	Describe population, secondary outcome
Activities of daily living	KATZ Activity of Daily Living Index	0,6,12	Describe population, secondary outcome
Impact on care-partners	Zarit Carer Burden Scale	0,6,12	Describe population, secondary outcome
Health-related quality of life	EQ-5D-5L	0,6,12	Describe population, secondary outcome and analysis of economic efficiency
Other adverse events	Number and event type (Recorded in falls diary for the last 6-months)	6,12	Describe population, secondary outcome
Program implementation cost	Customised survey: • Implementation and running of physical activity and dementia referral pathway, training of physical activity personnel and participant costs for program access.	1,2,3,4,5,6,7,8,9,10,11,12	Describe population and analysis of economic efficiency
Cost data (for the person with dementia)	Customised survey: Support from informal and formal care-partners, health care utilisation, pharmaceuticals, and opportunity costs. • The person living with dementia (with support from their care-partner) will be asked to recall support and health care utilisation over the last 6-months	0,6,12	Describe population and analysis of economic efficiency
Effect cost	• Falls and fall injuries (only for the person living with dementia) • Quality of life (both the person living with dementia and their care-partner)	0,6,12	Describe population and analysis of economic efficiency
Outcome of the shared decision process	• Type and amount of physical activity per week they currently do • Physical activity program(s) the person (and/or the care-partner if applicable) decided to take part in • Physical activity goal • Factors influencing the person’s choice	0	Describe population and guide the physical activity program, referral pathways and ongoing support required
Review of physical activity program the person is undertaking	Open text	6,12	Describe population and guide the physical activity program, referral pathways and ongoing support required
Review of physical activity goal/s that were set	Open text	6,12	Describe population and guide the physical activity program, referral pathways and ongoing support required
Achievement of physical activity goal/s relative to their expectation	Categorical	6,12	Describe population and guide the physical activity program, referral pathways and ongoing support required
***For the care-partner of the person with dementia***			
**Construct**	**Measure**	**Time points (months)**	**Purpose**
Age	Date of birth	0	Describe population
Gender	Categorical	0	Describe population
Relationship to the person	Categorical	0	Describe population
Living with the person	Yes/No	0	Describe population
Years caring for the person	Years	0	Describe population
Past experience with assisting the person with physical activity participation	Yes/No	0	Describe population
Physical activity program/s they have helped the person with	Open text	0	Describe population
Health related quality of life	EQ-5D-5L	0,6,12	Describe population and analysis of economic efficiency
Program acceptability	Is the “*Changing the Focus*” program suitable for you as a care-partner? • Yes/no/unsure Is the “Changing the Focus” program adequate to meet your needs as a carer? • Yes/no/unsure	6,12	Primary outcome
Program acceptability	Semi-structured interview	6 (or at the time of withdrawal)	Primary outcome for implementation evaluation
***For referrers of the person with dementia participating in “Changing the Focus”***			
**Construct**	**Measure**	**Time points (months)**	**Purpose**
Program acceptability	Semi-structured interview	1–2	Primary outcome for implementation evaluation
***For physical activity providers of the person with dementia participating in “Changing the Focus”***			
**Construct**	**Measure**	**Time points (months)**	**Purpose**
Program acceptability	Semi-structured interview	6	Primary outcome for implementation evaluation

**Table 2:** Description of assessments and methods for effectiveness measures for people living with dementia.

Assessment	Assessment domain	Description of method
**Primary effectiveness outcome**		
Active Australia Survey	Physical activity (mins / week)	The Active Australia Survey is a brief eight question survey that assesses participation (frequency and duration) in various types of moderate and vigorous activities in the preceding week [35]. The survey is a short and reliable set of questions that can be administered through telephone or face-to-face interviews (for this study the survey will be administered face to face in the home visit assessments). If the preceding week was not a typical week for the person’s physical activity (e.g. they were unwell), then the questionnaire is completed for the most recent typical physical activity week in the month preceding the assessment.
**Secondary effectiveness outcomes**		
Life Space Assessment-Cognitive Impairment questionnaire		A modified version of the Life-Space assessment tool for use with people with cognitive impairment. It assesses frequency of mobility in six life-space zones (within the 1. bedroom; 2. home; 3. Immediate surroundings of home; 4. local neighbourhood; 5. home town; and 6. unlimited), and assistance needed to travel within each zone. Has good reliability, validity and change with intervention in samples of people with cognitive impairment [36, 37].
Step Test	Dynamic standing balance test	Assesses speed (number of completed steps) of rapidly stepping one foot fully on to and then off a 7.5cm high step within a 15 second period. Each leg stepping is assessed separately. The lowest score between the right leg and the left leg is reported. High reliability and validity have been reported, including for people with dementia [38, 39].
2-minute walk test	Measure of cardiovascular fitness or endurance	Assesses distance able to be walked in 2 minutes at comfortable, usual pace. High retest and inter-rater reliability have been reported, including in people with cognitive impairment [40].
30-second sit-to-stand test	Functional measure of leg strength	Assesses the number of times a person can stand up and sit down from an armless chair (approx. 45cm seat height) in 30 seconds (secs), with their arms folded across their chest. Has good reliability and validity, [41] and has been shown to improve with exercise in people with cognitive impairment [23].
Timed-Up-and-Go-test	Functional mobility	Assesses the time taken for a person to stand up from a seated position on a standard (approx. 45cm seat) chair, walk three metres to a marker on the floor at their usual pace, turn, return to the chair and sit back down in the chair. Has been shown to have good reliability and validity, including with people with cognitive impairment [39, 42].
Mini Mental State Examination	Cognition screening tool	An 11-item questionnaire (maximum score 30) that assesses a number of cognition domains including orientation, recall, and ability to follow simple commands [32].
Lubben Social Network Scale-6	Social isolation	An abbreviated 6-item version of the Lubben Social Network Scale to screen for social isolation. The tool assesses three questions that assess kinship relationships, and three questions that assess non-kinship relationships. The three questions relate to number of contacts at least once a month; number of kinship/non-kinship people close enough to be comfortable seeking help from; and number of kinship/non-kinship people that the person feels sufficiently at ease to discuss private matters. Scores range from 0–30 [43].
Physical Enjoyment Scale PACES-8	Physical activity enjoyment	An abbreviated 8-item scale asking respondents to rate how they feel about aspects of the exercises / physical activity being undertaken, under the overarching theme of enjoyment. Each item is rated on a 1–7 scale, with scores ranging from 8–56 [44].
KATZ Activities of Daily Living Index	Activities of Daily Living	Assesses independence across six functional activities of daily living, with activities rated as independent, able to perform but requires assistance (partially dependent), or dependent. Total scores range from 0 (dependence on all six activities) to 6 (independent on all six activities) [45].
EQ-5D-5L 39 (for both person with dementia and their care-partner)	Health-related Quality of Life	Assesses quality of life across five dimensions (mobility, self-care, usual activities, pain/discomfort, and anxiety/depression). Scores can be converted into a utility index [46, 47]. The tool also includes a EQ visual analogue scale. The EQ-5D-5L has been shown to capture health of people with cognitive impairment in terms of known group and convergent validity [48].

### Primary outcomes

1. Program participation. This will be assessed by the number of people living with dementia continuing with the program at 12-months (using >70% of participants continuing with their selected program(s) at 12-months as a benchmark for success).
2. Total physical activity time per week for the preceding week (or if the preceding week was atypical–e.g. the person was unwell and did not do physical activities, then the most recent typical week of physical activity in the preceding month will be documented). This will be measured by the Active Australia Survey [35] conducted at baseline, 6- and 12-months.
3. Program acceptability. This will be explored through a semi-structured interview with all participant groups (participants living with mild dementia and their care-partners, referrers and physical activity providers):
  - Participants with dementia and their care-partners will be interviewed at 6-months, or at the time of program withdrawal, using a purposive sample aiming for diversity of physical activity program and participation levels. The interview will explore their perspectives on acceptability of the program, the shared decision-making process, support for participation, perceived benefits of participation, barriers/facilitators to participation, and the future sustainability of the “Changing the focus” program.
  - Referrers will be interviewed at 1–2 months following providing one or more referrals, using a purposive sample covering for a diverse mix of health professionals such as general practitioners, physiotherapists, and exercise physiologists. The interview will explore how the referrer heard about the program, program acceptability from their perspective, the type of benefits they consider the person with dementia may obtain from increasing their physical activity, factors they consider are important in determining if a person with dementia might be suitable for participation in this type of program, how they may be able to support uptake and sustained participation in the program by the person living with dementia, and future sustainability of the “*Changing the Focus*” program.
  - Physical activity/exercise providers will be interviewed at 6-months, using a purposive sample for providers aiming for diversity of physical activity/exercise programs, and including those who have and have not participated in the optional training (described below) to support them working effectively with people living with dementia. The interview will explore physical activity providers’ satisfaction with the in-person training and/or online training resources if they undertook the optional training (training resources developed by the research team); suggested improvements that can be made to the training resources and any other training that they felt is required; their confidence with working with people living with dementia, and whether adjustments were required to accommodate the person living with dementia into the program; program acceptability from their perspective, perspectives about what worked well, what may have been able to be done differently to achieve better outcomes; changes noted associated with physical activity participation for the person with dementia involved in their program; and whether the exercise provider would be seeking opportunities to attract more people living with dementia to this type of physical activity program, and the future sustainability of the “*Changing the Focus*” program.

In addition, participants with mild dementia and their care-partners will answer a question at 6- and 12-month assessment time points “Is the “*Changing the Focus*” program acceptable (i.e. suitable for you and adequate to meet your needs)? They can select a response of “Yes”, “No” or “Unsure”. The study will use a target criterion of >75% as a benchmark to determine program acceptability.

### Secondary outcomes

A comprehensive suite of outcome measures to assess participants living with dementia at baseline, 6- and 12-months, will be used (see Table 1 describing all data items, time points, and purpose, and Table 2 for description of methods for each assessment). Where necessary, care-partners will assist in questionnaire completion. Outcome assessments include: (1) *Life space* using the Life Space Assessment-Cognitive Impairment questionnaire; [37] (2) *Dynamic balance* using the Step Test; [38] (3) *Endurance* using the 2-minute walk test; [49] (4) *Leg strength* using the 30-second sit-to-stand test; [50] (5) *Mobility* using the Timed-Up-and Go-test; [42] (6) *Cognition* using the Mini Mental State Examination; [32] (7) *Social connectedness* using the Lubben Social Network Scale-6; [43] (8) *Physical activity enjoyment* using the Physical Enjoyment Scale PACES-8; [44] (9) *Activity of Daily Living* using the KATZ Activities of Daily Living Index; [45] and (10) Health-related QoL using the EQ-5D-5L [46].

Care-partners will be evaluated on the *impact of caring* using the Zarit Carer Burden Scale, [51] as well as the EQ-5D-5L to assess *quality of life* at the three assessment time points [46].

Data for a number of other outcomes regarding implementation aspects that are aligned to the RE-AIM framework [29] will also be collected. The interviews with all participant groups will also be used to inform the RE-AIM domains (e.g. factors associated with maintenance). These outcomes include:

1. Reach. The project will be promoted through multiple avenues for recruitment, so reach will be limited to documentation by the research team about avenues targeted for recruitment, number of responses received through each avenue of recruitment, and proportion of those indicating interest who met study inclusion criteria and commenced participation in the study.

2. Maintenance. Sustained program participation and program attrition. This will be determined through documentation by the research team, and factors contributing to maintenance raised through the participant interviews.

3. Safety and additional physical activity time data. Safety (falls and other adverse events) and physical activity time for new programs/exercises undertaken after people living with dementia have commenced the “*Changing the Focus*” study will also be recorded by the participants and their care-partners. The World Health Organisation definition of a fall will be utilised: “inadvertently coming to rest on the ground, floor or other lower level, excluding intentional change in position to rest in furniture, wall or other objects” [52]. (page 1) At baseline, the person living with dementia will be asked to recall falls, and falls injuries, over the preceding 6-months. Then, for the following 12-months, they will complete a falls diary that will prospectively capture the number of falls, and injuries from falls. The falls diary data will be collected by the research therapist at each home visit and phone call.

4. Economic evaluation. An explorative economic evaluation will be undertaken as part of the implementation outcomes, to be reported according to the Consolidated Health Economic Evaluation Reporting Standards 2022 (CHEERS 2022) statement [53]. It will take a societal perspective, and will report the cost of program implementation, as well as the cost-effectiveness and cost-utility of the program, of the pre-post design *“Changing the Focus”* program.

The cost of program implementation analysis will include costs attributed to implementing and running the physical activity and dementia referral pathway, training of physical activity personnel, and participant costs for program access. Implementation costs of the *“Changing the Focus”* program will be collected via survey from the Program Manager on a monthly basis and will include the 6-months prior to program commencement to capture program development (e.g. developing the training program) and set-up costs (e.g. Information Technology and staff recruitment costs), as well as the 12-months of the program. This analysis will inform future program uptake and scaling in other regions.

Cost data for the person living with dementia that will be collected includes support from informal and formal care-partners, health care utilisation, pharmaceuticals, and opportunity costs. Cost data will be collected by the research therapist at the baseline, 6 and 12-month home visits. On each of these three occasions, the person living with dementia (with support from their care-partner) will be asked to recall support and health care utilisation over the preceding 6-months, using a modified version of a previously utilised cost data diary, [54, 55] which includes adjustments for recall bias [55, 56].

Effect data for the economic evaluation includes falls (for the person living with dementia) and quality of life (for both the person living with dementia and their care-partner: see Assessment and outcome measures: Secondary outcomes section). To assess quality of life, both the person living with dementia and their care-partner will be asked to complete the EQ-5D-5L quality of life tool at baseline, 6 and 12-months [46, 47, 57]. At each of the three timepoints, the raw scores will be converted into a utility index, and the change in utility index from one time point to the next will be used to calculate the quality adjusted life years (QALY) gained or lost [46, 47, 57]. Cost and effect data will then be combined for the cost-effectiveness analysis (costs and falls data only for the person living with dementia) and for the cost-utility analysis (costs and quality of life data for both the person living with dementia and their care-partner).

### Intervention

**Fig 3:**
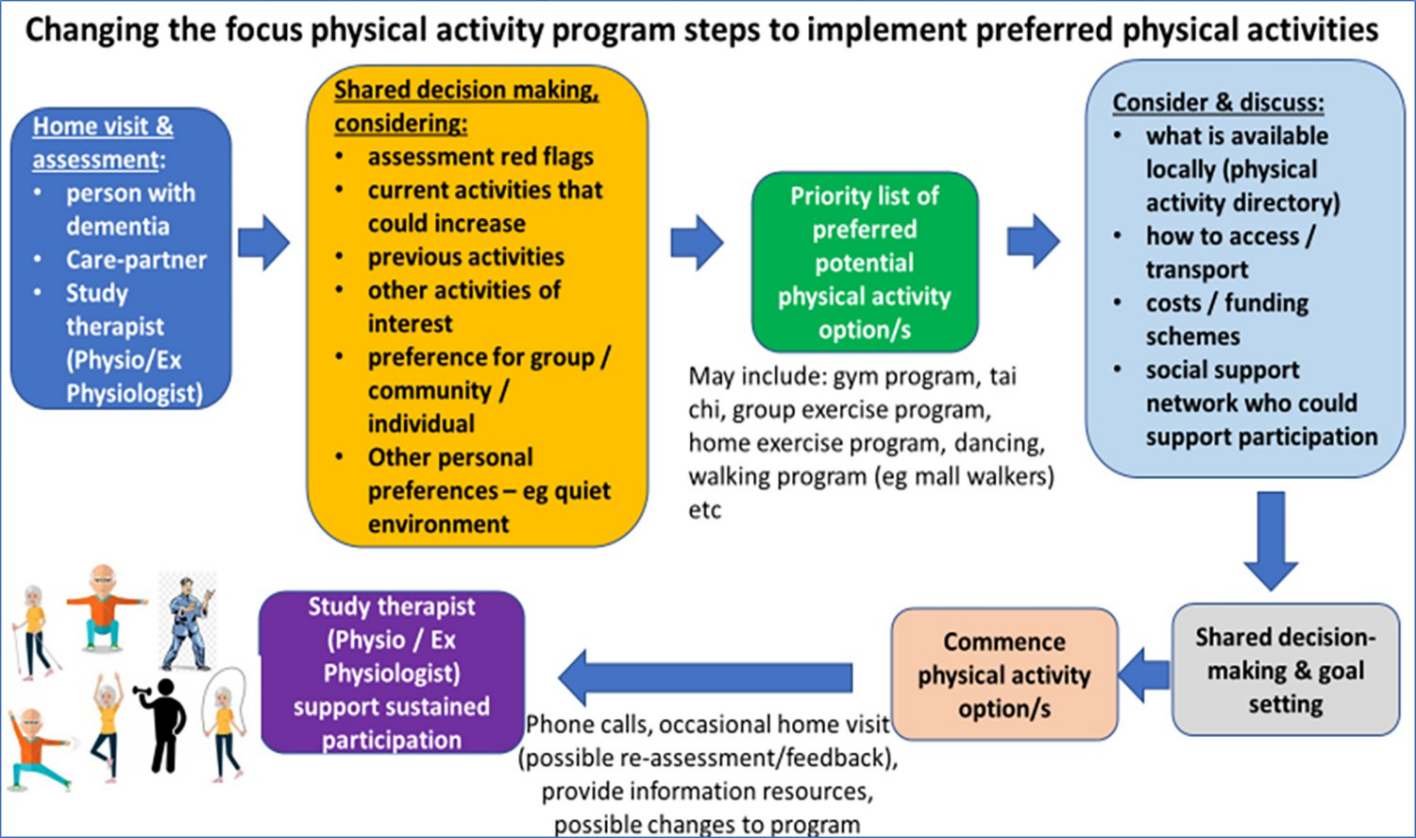
Steps in shared decision making to identify and implement preferred physical activity option/s.

**Fig 4:**
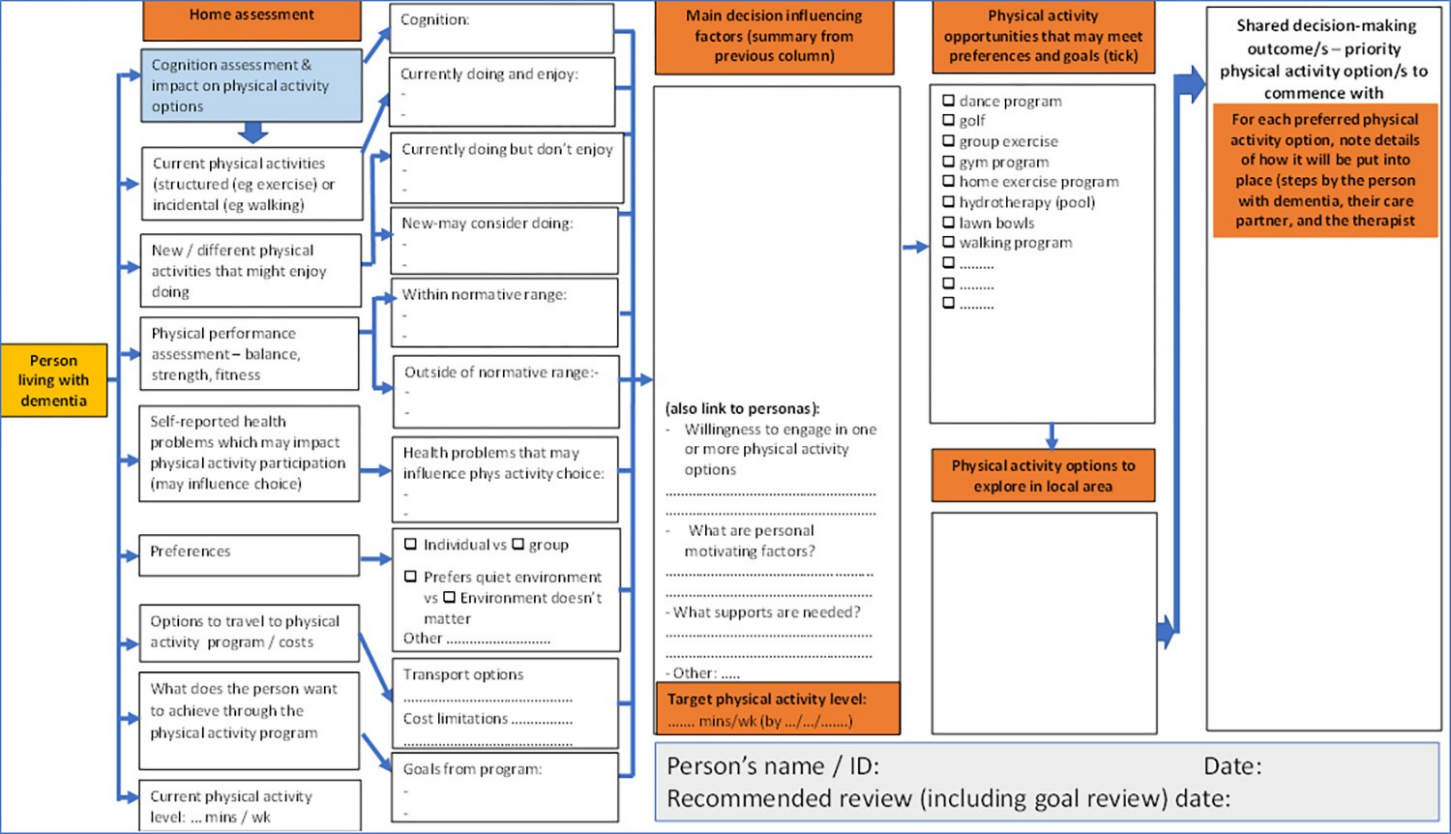
Shared decision-making support tool.

The “*Changing the Focus*” intervention is a 12-month program which will include the delivery of information and education-focused resources for health professionals and people living with dementia and their care-partners; exploration of referral pathways; completion of physical activity needs assessment; and supported implementation of targeted, individualised physical activity program/s to address the physical impairments and physical activity deficits and participatory goals of program participants. It will be underpinned by shared decision-making with the person with dementia and their care-partner (see Figs 3 and 4, and the Intervention procedures section), to improve and maintain function, independence, safety (reduce falls), and quality of life.

#### Development of a training program for physical activity providers

The project team will develop a no-cost optional training program particularly targeting physical activity providers in the recruitment regions who are interested in improving their understanding of strategies to support working with people living with mild dementia in programs they provide. The training program (an e-course) will include the following topics: (a) Welcome and an overview of the *“Changing the Focus”* program; (b) Module 1: Introduction to dementia; (c) Module 2: Changes in physical function and benefits of physical activity for people living with dementia; (d) Module 3: Physical activity and exercise considerations and ways for engagement for people living with dementia; (e) Module 4: Design considerations for making exercise facilities or environmental dementia-friendly; and (f) Module 5: Referrals for physical activity and funding options for people living with dementia.

While this training will be promoted to physical activity providers in the local region, and recommended to those who are providing physical activity for “*Changing the Focus*” participants, it will be optional for physical activity providers running physical activity option/s for “*Changing the Focus*” participants living with dementia. Some providers may already be confident in working with people living with dementia (e.g. having prior experience working with people living with dementia, or have undergone other related training previously). The physical activity provider training will include an in-person training session (approximately 2–3 hours in duration), highlighting the main parts of each module, delivered by the chief investigator and/or project staff in a group format at the commencement of the project recruitment. An online self-paced training program (approximately 2–3 hours) of all modules and further e-resources will be made available in the online training program, open to those interested throughout the study period. Another round of in-person training opportunities may occur approximately 6–9 months after project commencement, depending on the interest from new physical activity providers who engage with the program over time. Information brochures will also be developed or adapted from existing resources for people living with dementia and their care-partners about physical activity and participation.

#### Intervention procedures

**Fig 5:**
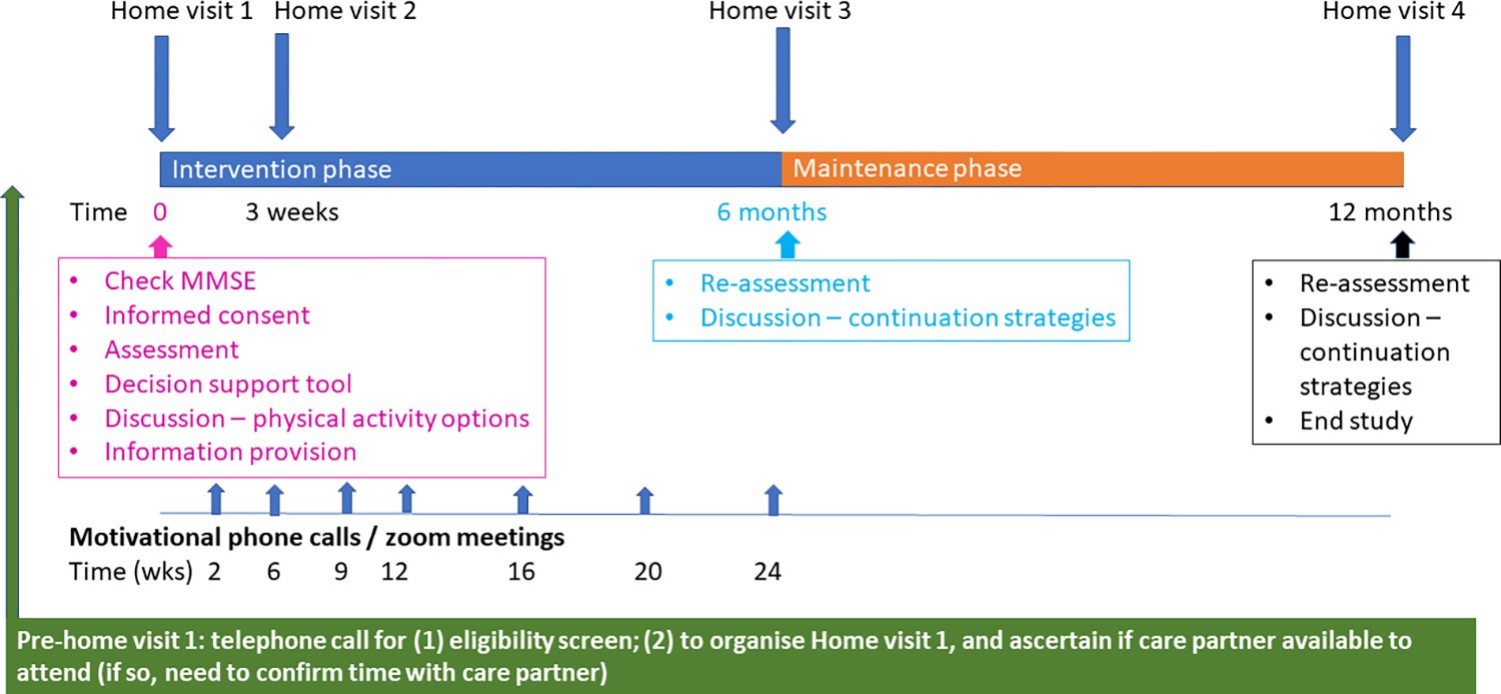
Participant study timelines.

The intervention will include four home-visits scheduled at baseline, week 3, 6- and 12-months, and seven motivational / support phone calls by the research therapist within the first six months (Fig 5). Additional home visits and phone calls may be arranged at the discretion of the research therapists if required to support program participation.

First home visit (~2.5 hours). In addition to the baseline assessment, the first home-visit by the research therapist will utilise a shared-decision support tool (Fig 4) to aid the decision-making discussion process with the person living with mild dementia and their informal care-partner (if applicable) to determine preferred physical activity options (Fig 3). This discussion will include (1) previous and current physical activity preferences they may enjoy, (2) identification of domains of physical performance (based on assessment outcomes) outside of age adjusted normal limits as potential intervention priorities, (3) cognitive and other health conditions that may influence physical activity participation, (4) discussion of physical activities that are likely to address impairments, (5) discussion of physical activity options available locally, along with related issues such as transport, access and cost, (6) one or more goals that the person living with mild dementia hopes to achieve through increased physical activity, and (7) setting of the initial (first month) physical activity time target for the participant.

At this home-visit, the research therapist will provide relevant information about commencing participation in the preferred one or more physical activities with the person living with mild dementia and their care-partner, and if necessary, help to initiate the participant’s commencement in the selected physical activity option/s. For example, this may include contacting the preferred physical activity provider to discuss the *“Changing the Focus”* program, the availability of the program’s face to face or online training, and factors from the baseline assessment and decision support process (with permission from the person living with dementia or their care-partner) that may be useful for the physical activity provider. All selected physical activity option/s for the participant’s program will aim to: (1) address identified physical impairments; (2) incorporate multimodal (balance, strength and fitness) physical activities tailored to the person’s needs, across the week; and (3) progress dosage of physical activity intervention by a nominal 10–20% per month from the baseline physical activity level, starting from the first month (e.g. if doing 30 min per week, initial target by end of first month will be 36 min per week) and aiming to progress each month towards the target of ≥150 min per week. Progression principles for increasing dosage will be provided to participants and care-partners, and discussed during motivational support calls during the 12-month intervention period. Participants will be able to continue with existing physical activities they have already been doing and add to these (by increasing dosage or adding a new physical activity) or to modify or replace one or more existing physical activities, based on the shared decision making and preferences and goals of the person living with dementia and their care-partner.

A local physical activity program directory (https://www.mornpen.vic.gov.au/Community-Services/Aged-Disability-Support/Positive-Ageing/Keeping-Active-Involved-and-Informed) will be made available to the research therapists who will support the decision making about suitable physical activity options in the local area for each participant living with mild dementia, as well as to referrers and physical activity providers who request a copy. Examples of local physical activity programs that may be discussed include group exercise activities (e.g. community health centre exercise programs, Council on The Ageing strength training programs, gym programs, and walking programs). Individually tailored home exercise programs will also be offered as another option. If selected through the shared decision-making process, these will be developed and delivered by the research therapists, and be supported by telehealth / intermittent home visit delivery options.

Second home visit (approximately week 3, ~45 minutes). This home-visit by the research therapist will occur after the decision-making session and when the physical activity plan has commenced, to discuss progress and participation in the physical activity program, and to provide motivational support.

Third home visit (at 6-months, ~1 hour). This home-visit by the research therapist will coincide with the 6-month assessment. At this visit, the research therapist will: (i) review physical activity goals and program(s) to determine if they are still meeting the needs of the participant or needing a change, (ii) repeat physical performance and other outcome measures (see Assessment and outcome measures: Secondary outcomes section), and discuss (iii) participation level, (iv) participation barriers and facilitators, and (v) maintenance of physical activity longer term.

Fourth home visit (~1 hour). This home-visit by the research therapist will coincide with the 12-month assessment. As well as completing the 12-month assessment, the research therapist will discuss: a) participation level, b) participation barriers and facilitators, and c) maintenance of physical activity longer term, following the end of the *“Changing the Focus”* program.

Motivational support (~20 minutes for each phone/video call). After the first home-visit, motivational support will be provided by the research therapist through regular telephone contacts. Seven phone calls (or video calls) will occur in the first 6-months of each participant’s intervention (suggested phone schedule at week 2, 6, 9, 12, 16, 20 and 24). Guided by Michie et al.’s COM-B framework for behaviour change, [58] the aim is to increase Capability (C, defined as the individual’s psychological and physical capacity to engage in the physical activity program by having the necessary knowledge and skills), capitalise on the Opportunity (O, defined as all the factors that lie outside the individual that make physical activity participation possible) to reinforce exercise behaviour provide, and to support Motivation (M, defined as processes that energise and direct behaviour including goals and decision-making).

#### Intervention fidelity checking

Intervention fidelity checking will be undertaken by collecting data throughout the project as to whether each component of the *Changing the Focus”* program is completed as planned, via: (i) self-report by people living with mild dementia and care-partners, and (ii) allocation of ratings by research therapists.

#### Communicating important protocol modifications

Protocol variations approved by the project team will be submitted for Ethics Committee amendment approval. Approved protocol variations will be recorded on the Trial Registration, and will be reported in the publication of study results.

### Sample size consideration

A formal sample size calculation is not relevant for this feasibility study. However, we have considered our sample size based on the main primary outcome of program participation (benchmark criterion for success >70% of participants with mild dementia continuing with “*Changing the Focus*” at 12-months). Using a sample size of 60 people living with mild dementia, we will be able to estimate a participation rate of 71% to within a 95% confidence interval of +/- 11.5%.

The sample size for the interviews of people living with dementia and their care-partners, referrers and physical activity providers (estimated to be 15–20 per group), will each be to the point of adequate information power [59].

### Data management and analysis plan

All assessment data (primary and secondary outcomes) collected at baseline, 6- and 12-months will be entered into a data file stored on a secure drive on Monash University’s server by a member of the research team. Qualitative data from interviews will also be stored on this server in computer data files as audio/video recordings and transcripts. Any information obtained in connection with this study will be made anonymous after data analysis is completed. Access to data files will be restricted to Chief Investigators who have log-on and a secure password to the data files, following approval from project lead investigators (KH, DCAL). The storage and the destruction of hard copies data file and hard copy data will adhere to Monash University regulations. The final report, and any resulting conference, journal publications or presentation will only contain results from participant responses that are aggregated or individual responses de-identified.

Descriptive analyses will be used to report demographic characteristics and the primary outcomes of participation and program acceptability based on the >70% and >75% benchmark criterion of success respectively as well as other outcomes regarding implementation aspects (i.e. reach, maintenance, safety and additional physical activity time data). Adverse events including falls and injuries will be summarised using descriptive statistics and narratives.

Interview data regarding program acceptability and other implementation outcomes from all participant groups will be recorded and transcribed verbatim, with two researchers independently reviewing and coding data to generate themes and sub-themes using thematic analysis [60].

For the primary outcome of total physical activity time per week in the last week and secondary outcomes, a general linear mixed model [61] using fixed effects for the set time points, random effects for the participants will be used to analyse the change in outcomes over time. In the case of missing data for the physical performance tests due to “inability to perform” e.g. physical limitations of the participants, we will assign a value (indicating the worst possible score) to the missing data for this reason and use ordered logistic regression instead. The assigned value would be different for these tests. For the dynamic balance step test, 2-minute walk test and the 30-second sit-to-stand test, the assigned number will be zero, while the Timed-up-and-Go-test would be 999 (assuming no participant who actually completes the test has a higher value than this).

#### Analysis of economic evaluation

The costing of items in the economic evaluation will be based on actual costs where available, and where these are not available, costs (excluding care-partner time) will be based on market rates, with care-partner time based on the current Australian minimum wage. All costs will be presented as AUD 2024/25, with costs collected prior to 2024/25 to be inflated by the consumer price index (https://www.rba.gov.au/calculator/).

The cost of program implementation analysis will report each cost type (including costs attributed to implementing and running the physical activity and dementia referral pathway, training of physical activity personnel, and participant costs for program access), including the number of units utilised, the cost per unit, and the total cost. Results will be presented as an overall cost, as well as a cost per participant to inform the cost-effectiveness and cost-utility analyses.

Cost data will be collected for the person living with dementia and this will be analysed for each time point using an independent t-test with the mean (SD) reported, as well as the mean difference (95%CI) between time points.

Effect data includes falls (only for the person living with dementia) and quality of life (both the person living with dementia and their care-partner). EQ-5D-5L raw scores will be converted into a utility index, and the change in utility index from one time point to the next will be used to calculate the quality adjusted life years (QALY) gained or lost [46, 47, 57]. Effect data will be analysed for each time point using an independent t-test with the mean (SD) reported, as well as the mean difference (95%CI) between time points.

Cost and effect data will be combined for the cost-effectiveness analysis (costs and falls data only for the person living with dementia) and for the cost-utility analysis (costs and quality of life data for both the person living with dementia and their care-partner). The incremental cost effectiveness ratio (ICER) will be calculated using the bootstrap method (5,000 replications) with the results presented on a cost-effectiveness plane and as a probability of cost-effectiveness across a range of willingness to pay thresholds (AUD $0 to $50,000) [62].

### Dissemination of study findings to participants

A plain language summary (de-identified) will be provided on the Rehabilitation, Ageing and Independent Living (RAIL) Research Centre website and forwarded to all participants at the end of the project.

## Discussion

With growth in ageing populations and the proportion of older people living with dementia globally, there is an urgent need for approaches that can reduce the rate of decline in physical and mental health and wellbeing, or even improve some outcomes, for people at the mild stage of the dementia trajectory. Increased physical activity shows promise as an approach that may achieve these outcomes [19–21, 23]. Achieving increased physical activity may sustain or improve independence and increase ability to remain living at home, and possibly delay need for some community services or residential care. It may also potentially reduce the high levels of care-partner stress for care-partners of people living with dementia [63].

However, there is no current systematic approach to support uptake and sustained engagement of physical activity for people living with dementia. The “*Changing the Focus*” program is a novel approach involving key stakeholders that has potential to influence physical activity behaviour change for people living with mild dementia, in a real-world setting (utilising mainly existing physical activity programs). The program has been developed with co-design with key stakeholders. A critical component of the approach of the “*Changing the Focus*” program is the shared decision-making process, using a tool specifically developed for this purpose (Fig 3). Shared decision-making has been previously utilised to optimise relevance and participant choice to achieve higher levels of uptake and sustained participation in interventions, including with people living with dementia, [64] and can support people living with dementia feeling empowered [65] and engaged in their health promotion choices [66].

In a real-world setting, the appropriate attitudes, knowledge, confidence and skills of physical activity providers regarding dementia, and possible strategies that may need to be considered for their programs to meet the needs of people living with dementia, are also critical. Recent research has shown that many health professionals have gaps in their dementia knowledge and confidence [67–69]. The availability of training for physical activity providers in the “*Changing the Focus*” program, including the provision of both in-person and on-line modules, aims to support physical activity providers to work effectively with people living with dementia so that they are more able to participate and be included in mainstream community physical activity programs.

The shared decision making and motivational support, and optional training program for physical activity providers through this proposed intervention provide a framework with potential for embedding a sustainable behaviour change for enjoyable and meaningful physical activity option/s for the person living with dementia. Feasibility outcomes, qualitative and economic evaluation data will inform and guide future large-scale studies to determine program effectiveness and cost-effectiveness. These will contribute to innovative approaches for enhancing physical activity opportunities for this population.

## Supporting information

S1 Checklist The TIDieR (Template for Intervention Description and Replication) checklist*: Information to include when describing an intervention and the location of the information. (DOCX)

S1 Appendix Cognitive capacity checklist. (DOCX)
