# Supplementary material for: Changing the focus: Facilitating engagement in physical activity for people living with mild dementia in a local community—Protocol for a pre-post mixed methods feasibility study
Source: PLoS One. 2024 Sep 13;19(9):e0307018. doi: 10.1371/journal.pone.0307018 (PMC11398674; doi:10.1371/journal.pone.0307018)
Supplement: S1 Appendix — (DOCX) [file pone.0307018.s002.docx]

**Supplementary File 1.**

Evaluation to Sign Consent

This form is to be used to ascertain a person’s ability to provide informed consent for themselves. This evaluation is ideally conducted face to face. If not possible, alternatives such as online videoconference or telephone will be possible but are not ideal.

A person may be unable to provide consent due to a disease process, such as dementia or a mental health condition, or due to structural disadvantage, such as low literacy level or English as a second language.

**Please tick the applicable boxes**

Participant Information and Consent Form (PICF) previously received in written form

Yes □ No □ Not applicable □

Previous discussion with participant proxy to gain some understanding of whether they feel the person with dementia will be able to consent, and have been provided an idea of their level of dementia

Yes □ No □ Not applicable □

Explanation of project given (by reading or paraphrasing PICF)

Yes □ No □ Not applicable □

Participant alert and able to communicate

Yes □ No □ Not applicable □

If unable to be conducted face to face, has the proxy been provided the questions in advance and informed that you will call back later at a convenient time when they can speak freely

Yes □ No □ Not applicable □

**For the evaluation:**

1. Questions must be answered correctly
2. The questions can be rephrased to ensure that the participant understands what you are asking.
3. If the person answers with “I don’t remember”, you can read the section again.
4. Participants should not merely repeat what you are saying, they must respond in their own words to ensure understanding.
5. If the participant does not successfully answer all the questions correctly, then proxy consent is needed, but best practice states that assent and (lack of) dissent should be sought.

| **Question** | **Acceptable answers (adapt to be relevant to the project)** | **Answered successfully**  **(yes/no) (or N/A for question 5)** |
| --- | --- | --- |
| 1. What do you understand the project is about? |  |  |
| 1. What is expected from you, the participant? |  |  |
| 1. What are the potential risks? |  |  |
| 1. What if you don’t want to continue? |  |  |
| 1. What if you experience discomfort? |  |  |
| 1. What are the potential benefits of taking part? |  |  |

If there is a “no” to any of these or are uncertain about any of these, do not seek written consent from the person. Instead, you (the project physiotherapist) will need to:

1) ascertain if the person has either or both of the following:

- Advanced care directive (check that the research does not contravene this)
- A medical treatment decision maker

Informed consent will be sought from this medical treatment decision maker if there is an appointed medical treatment decision maker. If not, their informal carer will provide informed consent on behalf of the person. The informal carer is the first of the following with a close and continuing relationship with the person:

• The spouse or domestic partner

• The primary carer of the person

• The oldest adult child of the person

2) explain the program to the research decision maker and ensure their understanding

3) seek informed consent from the research decision maker on behalf of the person before proceeding with the program.

**Reference:** Resnick, B., et al. (2007). "Reliability and validity of the Evaluation to Sign Consent measure." The Gerontologist 47(1): 69-77.
